# Less spatial exploration is associated with poorer spatial memory in midlife adults

**DOI:** 10.3389/fnagi.2024.1382801

**Published:** 2024-06-11

**Authors:** Vaisakh Puthusseryppady, Daniela Cossio, Shuying Yu, Farnaz Rezwana, Mary Hegarty, Emily G. Jacobs, Elizabeth R. Chrastil

**Affiliations:** ^1^Department of Neurobiology and Behavior, University of California, Irvine, Irvine, CA, United States; ^2^Department of Psychological and Brain Sciences, University of California, Santa Barbara, Santa Barbara, CA, United States; ^3^Neuroscience Research Institute, University of California, Santa Barbara, Santa Barbara, CA, United States

**Keywords:** spatial exploration, spatial navigation, spatial memory, age, virtual reality, midlife

## Abstract

**Introduction:**

Despite its importance for navigation, very little is known about how the normal aging process affects spatial exploration behavior. We aimed to investigate: (1) how spatial exploration behavior may be altered early in the aging process, (2) the relationship between exploration behavior and subsequent spatial memory, and (3) whether exploration behavior can classify participants according to age.

**Methods:**

Fifty healthy young (aged 18–28) and 87 healthy midlife adults (aged 43–61) freely explored a desktop virtual maze, learning the locations of nine target objects. Various exploration behaviors (object visits, distance traveled, turns made, etc.) were measured. In the test phase, participants navigated from one target object to another without feedback, and their wayfinding success (% correct trials) was measured.

**Results:**

In the exploration phase, midlife adults exhibited less exploration overall compared to young adults, and prioritized learning target object locations over maze layout. In the test phase, midlife adults exhibited less wayfinding success when compared to the young adults. Furthermore, following principal components analysis (PCA), regression analyses indicated that both exploration quantity and quality components were associated with wayfinding success in the midlife group, but not the young adults. Finally, we could classify participants according to age with similar accuracy using either their exploration behavior or wayfinding success scores.

**Discussion:**

Our results aid in the understanding of how aging impacts spatial exploration, and encourages future investigations into how pathological aging may affect spatial exploration behavior.

## Introduction

Spatial navigation—the ability to determine and maintain a trajectory from one location to another—is a fundamental cognitive ability that we use in our daily lives (Gallistel, 1990). Spatial navigation can broadly be broken down into two aspects—spatial *exploration* and spatial *memory*. Spatial exploration refers to the navigation patterns a person exhibits as they investigate and learn a novel spatial environment (i.e., a process used by people to form new spatial representations), while spatial memory refers to one's stored spatial representations of environments, which are subsequently used to help them navigate within those environments (Wolbers and Hegarty, 2010; Johnson et al., 2012). As a highly complex behavior, spatial navigation exhibits variation in the human population (Newcombe, 2018; Newcombe et al., 2022; Hegarty et al., 2023). Factors that may contribute to this variation include age, sex, childhood environment, neurodegeneration, and general cognitive ability, to name a few (Pu et al., 2020; Yu et al., 2021; Coutrot et al., 2022; Puthusseryppady et al., 2022). In particular, the effect of age on spatial navigation has been a widely studied topic in the field.

The majority of studies investigating the effect of age on spatial navigation have focused on spatial memory—the subsequent knowledge and representations of space after learning. The overarching findings of these studies suggest that various aspects of spatial memory decline with normal aging. Changes have specifically been reported in the ability to use and switch between allocentric (world-centered, viewer independent) and egocentric (self-centered, viewer dependent) reference frames, landmark knowledge (binding of landmarks to directional information), and path integration (continuously yielding updated estimates of position and orientation in space) (Harris and Wolbers, 2012; Lithfous et al., 2013; Lester et al., 2017; Zhong and Moffat, 2018; Wiener et al., 2020; Zhang et al., 2021). These changes have been mainly attributed to alterations in the structure and function of the brain's navigation network that naturally occur with age. In terms of structure, studies have suggested the age-related loss of gray matter volume in the hippocampus, prefrontal cortex, and caudate nucleus to underlie age-related declines in spatial memory (Moffat and Resnick, 2002; Driscoll et al., 2003; Moffat et al., 2007) while in terms of function, these declines have been attributed primarily to reduced activations in the hippocampus (Moffat et al., 2006; Konishi and Bohbot, 2013). Meanwhile, a set of studies investigating changes to navigation during pathological aging have suggested that impairments to spatial memory could be an early cognitive marker for Alzheimer's Disease (AD) (Kunz et al., 2015; Allison et al., 2016; Coughlan et al., 2019). Allocentric reference frames are particularly affected, owing to their reliance on medial temporal lobe structures such as the hippocampus and entorhinal cortex (Moffat et al., 2006), which are among the very first brain regions affected by the AD pathology (Braak and Braak, 1995). Some studies have focused on assessing spatial memory abilities in midlife—a critical period early in the aging process where cognitive changes begin to emerge (Dohm-Hansen et al., 2024). The results from these studies suggest that declines to spatial knowledge acquisition (i.e., landmark knowledge, understanding the connectivity of different locations in an environment, or drawing a map of the environment) and changes to navigation strategy use are evident by midlife. In contrast, some abilities like path integration and route knowledge seemed to be spared (Jansen et al., 2010; Zhong and Moffat, 2016; Yu et al., 2021).

Compared to the extensive research conducted on spatial memory and aging, very few studies have investigated how spatial exploration behavior changes with age. Most studies on this topic have been conducted on rodents, and the overarching findings of these studies show that when compared to young rodents, aged rodents exhibit less exploration of an open field (Glenn et al., 2008; Adelöf et al., 2019) and of novel arms in a maze (Lamberty and Gower, 1990). These findings of age-related reductions in exploration seen in rodents have also been seen and reported in other animals, such as wasps (Thiel et al., 2006) and fish (Yu et al., 2006).

Human studies of exploratory behavior to date have mainly focused on young populations, investigating how exploration behavior varies according to sex in healthy young adults (Gagnon et al., 2018; Munion et al., 2019) or in children with or without developmental disabilities (Mandolesi et al., 2009; Farran et al., 2022). In particular, studies in the healthy young adult population have highlighted an association between exploration patterns and subsequent spatial memory performance in both virtual reality (VR) and real-world environments. These studies suggest that sex differences in exploration patterns explain sex differences in subsequent spatial memory. Specifically, when compared to females, male advantages in the ability to navigate and point to target locations within an environment was mediated by less revisiting of previous locations, higher rates of spreading through an area, pausing less, and traveling further distances without changing course during free exploration of this environment (Gagnon et al., 2018; Munion et al., 2019). Another study showed that spending more time exploring paths that have high global connectivity within a virtual town environment lead to better subsequent cognitive map formation of this environment (Brunec et al., 2023). Lastly, the choice of using one of two distinct exploration strategies when learning object locations in a room (i.e., exploring along the main axes of the room or in a circular pattern around the edges of the room) had an impact on how efficiently people navigated to the objects in a subsequent search task (Kallai et al., 2005). Specifically, the use of a circular exploration strategy was associated with more efficient navigation, as measured by shorter path lengths.

Studies investigating exploration behavior in older populations have focused specifically on visual exploration (i.e., eye movement patterns). The results of these studies show older adults as being more spatially selective in their visual exploration of a novel spatial environment, for example, they choose only a single set of windows (out of a total two sets) to view and explore a room (Varner et al., 2016). Likewise, older adults demonstrate a specific focus on learning the relationships between local objects and external landmarks as opposed to learning the global layout of the objects (Segen et al., 2022). Increased age in this population was also associated with longer reaction times and total eye saccade times on a visuospatial figure matching task (Kaneko et al., 2021). Two other studies, which investigated exploration behavior in the context of a virtual foraging task, reported older adults as exhibiting less exploratory behavior (i.e., less coverage of the environment) by staying longer and foraging for resources in a chosen region of the environment, as opposed to foraging in other regions of the same environment (Mata et al., 2009; Louâpre et al., 2010).

Although a few studies have investigated spatial exploration in aging, they have not studied this behavior in detail in midlife, nor in the context of learning and navigating within novel spatial environments. To the best of our knowledge, the only findings to date are preliminary results from a study conducted by our team (which utilized a subset of the participants used in the current study) showing that when compared to younger adults, midlife adults made fewer button presses on a keyboard, which controlled exploration through the maze (Yu et al., 2021). Hence at present, we do not understand the extent of details to which navigation patterns while exploring and learning a novel spatial environment change in midlife, and how this change might in turn influence spatial memory for that environment.

From a behavioral perspective, findings from previous studies have shown that age-related changes to spatial exploration can be seen in rodents, wasps, and fish (Thiel et al., 2006; Yu et al., 2006; Adelöf et al., 2019), and that with increasing age, species including humans exhibit lower tendencies to engage in explorative (as opposed to exploitative) behaviors (Eliassen et al., 2007; Wang et al., 2009). Furthermore from a neural perspective, findings from previous studies have suggested active spatial exploration to be attributed to activity in the medial temporal lobe structures (i.e., hippocampus, entorhinal cortex)—regions that are highly sensitive to age effects (Driscoll et al., 2003; Song et al., 2005; Winter et al., 2015). Thus, taken together, we expect age-related changes to exploration behavior to be seen in the context of learning a novel spatial environment. Considering that notable structural changes to the hippocampus and entorhinal cortex occur as early as midlife (Fjell et al., 2013; Hasan et al., 2016), we expect to see this manifest behaviorally as changes to exploration behavior in the midlife period.

Here, we investigate changes to exploration behavior of a virtual maze environment and its impact on the formation of spatial graph knowledge (i.e., understanding of the connectivity between different maze locations) in midlife adults. For assessing exploration, we are particularly interested in looking at navigation behaviors representing amount of environmental coverage (e.g., pause duration, number of location visits, distance traveled, etc.), as these have been shown by previous studies to be related to spatial memory formation (Gagnon et al., 2018; Munion et al., 2019). We are also interested in assessing navigation behaviors denoting quality (i.e., spread) of environmental coverage. For example, Gagnon et al. (2018) found that increased spreading through an area during exploration was related to better spatial memory formation. Although Brunec et al. (2023) did not find a relationship between path roaming entropy and spatial memory, they did find that those with better spatial memory spent more time exploring particularly informative areas of the environment. We also think evenness of exploration could be an important variable to consider as intuitively, it can be reasoned that sampling different regions of the environment to a similar degree can contribute to better spatial memory formation for that environment.

Investigating age-related changes to spatial exploration behavior is important because it can further our theoretical understanding of the effects of normal aging on spatial navigation across the lifespan, generating knowledge which can in turn be used to help develop cognitive markers to aid in the early identification of pathological aging (e.g., AD). Furthermore, studying the relationship between spatial exploration behavior and spatial memory can potentially offer avenues to improve individuals' spatial memory by means of encouraging them to alter their exploration behavior.

To this end, the main aim of this study is to investigate spatial exploration behavior in healthy midlife adults, as they learn and navigate in a novel spatial environment. By using a desktop virtual reality spatial environment, we specifically aimed to assess:

i) Whether and how spatial exploration behavior is altered in healthy midlife adults when compared to healthy young adults;
ii) The relationship between spatial exploration behavior and subsequent spatial memory in both midlife and young adults; and
iii) Whether measures of spatial exploration behavior represent a more sensitive marker for cognitive aging compared to spatial memory performance.

We hypothesized the following. First, alterations to spatial exploration behavior will be seen in midlife adults when compared to the young, building on preliminary findings from our previous study showing less exploration behavior in midlife adults (Yu et al., 2021). Furthermore, with previous studies reporting less exploration with age across species (e.g., rodents, wasps, fish) (Thiel et al., 2006; Yu et al., 2006; Adelöf et al., 2019), we hypothesize that this pattern may also be seen in humans during midlife, owing to changes seen in the medial temporal lobe structures in this period (Fjell et al., 2013; Hasan et al., 2016). Second, more extensive spatial exploration behavior (i.e., quantity and quality of environment coverage) will be associated with better spatial memory in both the midlife and young adult groups, following similar relationships previously reported in healthy young adults (Gagnon et al., 2018; Munion et al., 2019). Third, we will be able to classify our participants into young or midlife more accurately using their spatial exploration behavior when compared to their spatial memory, reflecting exploration as a relatively more sensitive marker for cognitive aging. This result would be in line with findings from a previous study showing age-related differences in visual exploration patterns but not in spatial memory (Segen et al., 2022). Here, we expect the same pattern to be seen in with regards to physical exploration. Alternatively, we could also expect that both exploration behavior and spatial memory will be able to classify our participants into young or midlife categories with similar accuracy, hence representing equally sensitive markers for cognitive aging. This result is possible because, intuitively, it can be argued that age-related alterations to the spatial learning process could directly result in worse spatial memory.

## Materials and methods

### Participant recruitment

A total of 109 healthy midlife adults (mean age = 50.65 years ± 3.68, 74 females and 35 males) and 51 healthy young adults (mean age = 19.47 years ± 1.28, 26 females and 25 males) were recruited for this study from the University of California, Santa Barbara and the greater Santa Barbara region. The relatively larger sample size for the midlife adults is due to these individuals being recruited for another large study on the impact of menopause on navigation ability. Inclusion criteria for the participants were being able to understand English, having no visual impairments, and being able to perform simple key presses with their fingers. Exclusion criteria were having a history of (or current) neurologic disease (i.e., dementia, stroke, epilepsy, migraine, head trauma, etc.), psychiatric illness, or current use of psychotropic medication. Signed informed consent was obtained from all participants prior to undergoing the experimental protocol, and the participants were compensated financially for their study participation (young adults at $12 per hour or course credit; midlife adults at $30 per hour). Ethical approval for this study was obtained from the University of California, Santa Barbara Human Subjects Committee.

Due to technical issues with the equipment and/or motion sickness, some participants could not complete all 24 trials of the Maze Learning Task (see below) resulting in the data for 1 young participant and 5 midlife participants being discarded. Data from an additional 17 midlife participants, who completed all 24 trials, were also discarded due to technical errors in the recording of the data. Removing the respective participants resulted in a final sample size of 87 midlife (mean age = 50.27 years ± 3.70, 64 females and 23 males, mean years of education = 16.82 ± 1.84, AMNART^1^ Verbal IQ mean score = 125.48 ± 6.14, AMNART Performance IQ mean score = 118.18 ± 4.34, AMNART Full Scale IQ mean score = 124.68 ± 5.90) and 50 young participants (mean age = 19.44 years ± 1.28, 26 females and 24 males) to be used for all the analyses. A chi-square test of independence showed that there were differences in the distribution of sexes in the two age groups (X^2^ = 5.629, df = 1, *p*-value = 0.017), with the midlife group having a significantly higher proportion of females than the young group. Therefore, we controlled for sex by including it as a covariate in many of our analyses (see below).

### The maze learning task

#### Environment

**Figure 1:**
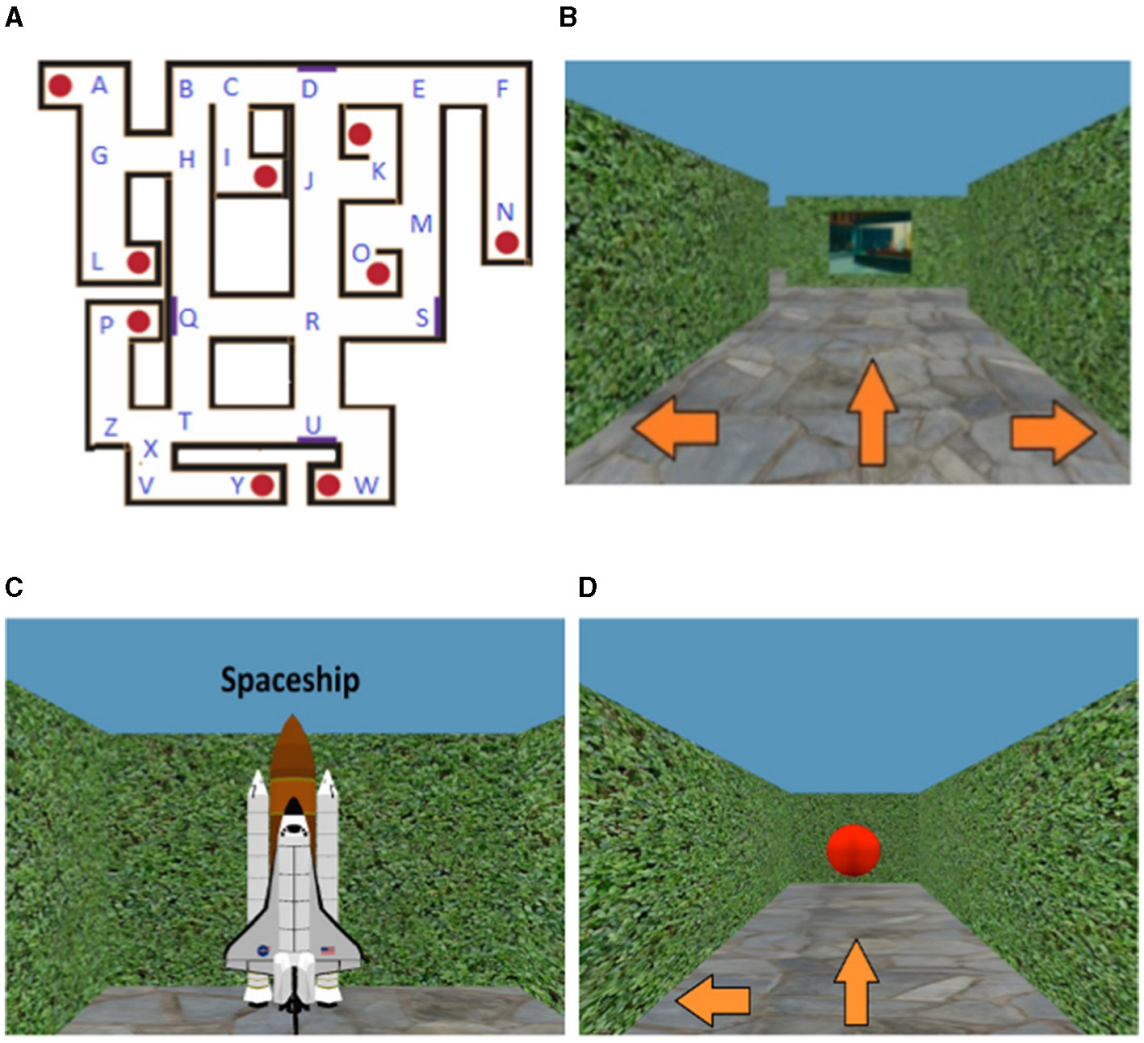
Overview of the Maze Learning Task. **(A)** Overhead map of virtual maze, with positions of target objects (red circles) and paintings (purple rectangles). All target object and hallway locations inside the maze have been respectively labeled with letters of the alphabet. **(B)** Participant view during maze navigation, with three possible directions to move (view of a painting ahead). **(C)** View of example target object (spaceship). **(D)** Participant view of target object during test phase—all target objects have been transformed into red spheres to minimize feedback. Figure modified from Yu et al. (2021).

All participants performed the Maze Learning Task using desktop virtual reality, on a 24-inch LCD monitor of a Dell Windows 7 computer. The Maze Learning Task assesses participants' ability to learn a novel spatial environment from unconstrained, free exploration, and to later navigate to learned locations within this environment. The details of this task have been provided in previous studies (Yu et al., 2021; Chrastil et al., 2022). In brief, the maze environment was composed of tall, vertical hedges that serve as walls, nine target objects positioned at various locations (guitar, snowman, spaceship, lamp post, chicken, trophy, chair, umbrella, cuckoo clock), four paintings that served as landmarks which participants could use to aid their orientation, as well as various hallways (Figure 1). Participants moved around in the maze using the keys on the keyboard. At each decision point (labeled “A–Z”—see Figure 1A), they indicated whether they wanted to go straight, left, or right using the corresponding arrow keys. Pressing the forward arrow key would move the participant to the next decision point straight ahead, while pressing the left/right arrow keys would make the participant rotate in place to face either left or right. The virtual motion to the next location/direction was visible to participants. In the maze, participants' translational movement was fixed at 1 virtual meter per second, and their rotation was fixed at 90° per second. An example of a test trial can be viewed here, which also demonstrates the virtual movement: https://www.youtube.com/watch?v=LMsGpo2Ss7M.

#### Exploration phase

In the Maze Learning Task, participants completed two different phases. The first was the exploration phase, which consisted of two consecutive exploration blocks of 8 min in duration. In each block, participants were instructed to freely explore the maze and learn the locations of the nine target objects.

#### Test phase

Following the exploration phase is the test phase, which consisted of a single block of 24 wayfinding trials. In each trial, participants were placed at a target object and asked to navigate to another target object within a given duration of 45 seconds. During this phase, all target objects were transformed into red spheres to minimize feedback and to prevent the use of potential landmark cues during their navigation. The main outcome measure for this phase was *wayfinding success*, which was defined as the proportion of successful trials (out of a maximum of 24).

Since the maze environment can be represented as a topological graph composed of place nodes (i.e., target object locations & hallway junctions) being connected by path edges (i.e., hallways), the ability to navigate from one target object to another requires the understanding of the connectivity of the environment (Tutte, 1984). This knowledge, termed as spatial graph knowledge, is the aspect of spatial memory that we consider the wayfinding success variable to be measuring (Chrastil and Warren, 2014).

### Data analysis

#### Exploration measures

For each participant, we obtained a single string sequence representing the temporal order of visited maze locations during each exploration block (e.g., AGHQR… see Figure 1A). Here, if a participant visits a specific location (i.e., target object or hallway junction) and turns in place (e.g., visits location A and turns in place to face away from A), this would be recorded as “AA,” with the first “A” representing the initial visit and the second “A” representing the turn in place. With some of our exploration measures of interest being based on counts of location visits, visits including turns in place (i.e., consecutive location repeats) were coded as a single visit for accuracy (AA becomes just A).

The exploration phase had 10 outcome measure variables, six of which are in line with previous studies that examined exploration behavior in humans (Meade et al., 2019; Ugwitz et al., 2019; Farran et al., 2022). *Distance traveled* is the total amount of ground covered in the maze (measured in virtual units), *turns made* is the total number of left or right turns in place made, *pause duration* is the total amount of time participants spent at each maze location before making their next move (measured in seconds), *button presses* is the total number of key presses made on the keyboard controller, and *target object visits* and *hallway visits* is the total number of visits made across all target objects and hallways, respectively. Three additional variables measure the distribution or spread of exploration (Gagnon et al., 2018; Brunec et al., 2023): *path roaming entropy, clustering of exploration of objects*, and *clustering of exploration of hallways*, which are described next. We were particularly interested in these variables due to the logical reasoning that the degree to which different regions of the environment are sampled relative to one another might influence the formation of spatial memories for the environment. *Path roaming entropy* is a measure of the probability of the participant being in a particular location in the maze during their exploration. To measure this, we first used each participant's exploration path sequences to calculate how often they visited each maze location; here, each maze location represents a bin. Next, we computed the Shannon's entropy for the distribution of maze location visits, across all bins, for each participant using the formula (Shannon, 1949):


$$\begin{array}{l} \text{H}=\;-\overset{n}{\underset{i=1}{\sum }}\text{P}\left(0_{i}\right)\;\log_{e}\text{ P}\left(0_{i}\right) \end{array}$$

Where *n* is the total number of bins, *i* is the current bin number, and P(0_i_) is the probability of a randomly selected location from the participant's exploration trajectory belonging to bin number *i*. Overall, higher path roaming entropy values indicate a high probability for the participant to be at any given location in the maze during their exploration (i.e., highly spread exploration) while lower path roaming entropy values indicate a low probability for the participant to be at any given location during their exploration (i.e., less spread exploration).

While path roaming entropy measures spread of exploration overall in the maze, the variables of *clustering of exploration of objects* and *hallways* particularly measure how spread the participants' target object and hallway visits were. To compute these variables, for each participant we first derived the distribution of their target object and hallway visits respectively, from which we then calculated their mean frequency of target object and hallway visits. The standard deviation of their distribution's mean was calculated, and this value was then used as our measure of clustering of exploration. Here, higher standard deviation values reflect more clustered exploration and lower standard deviation values reflect less clustered—or more even—exploration. Our final variable of interest is *longest hallway sequence*, which refers to the largest number of consecutive hallways visited without a target object visit. This variable provides us with a measure of the extent to which participants are learning the general layout of the maze hallways, rather than spending time learning the objects. In the context of the maze environment being represented as a topological graph, one can think of this variable as capturing the number of consecutive graph edges visited.

For each participant, we computed their values for all the above variables across both exploration blocks put together, and used these calculated values as the final scores.

#### Analysis steps

The data analysis was conducted in four steps, all in RStudio software package 2021.09.1 (Build 372). The anonymized study data can be found on OSF (https://osf.io/eskza/?view_only=909acb80078643a59d6ca4af68746409), and the R code used for data analysis can be found on Github (https://github.com/Vaisakh94/Midlife-Exploration-Manuscript.git).

##### Group differences in exploration and test phases

In the first step, we assessed group differences in each of the ten exploration variables and in the wayfinding success variable. Because of the unbalanced male and female ratios between the two age groups, for each variable we ran an ANCOVA with sex as a covariate. To run this analysis, all variables with a non-normal distribution were transformed into a normal distribution using either square root, inverse, or log transformations depending upon the magnitude of the skew. As exploratory *post-hoc* analyses, we assessed changes in the exploration variables from block 1 to 2 of the exploration phase in the midlife and young groups respectively. For both groups, we ran linear mixed models using the “*lme4*” (Bates et al., 2015) and “*lmerTest”* (Kuznetsova et al., 2017) packages in R, to control for the variable of sex. For each model, block number and sex were input as the fixed effects, participant number as the random effect, and each respective exploration variable as the dependent variable. Effect sizes for the above ANCOVAs and linear mixed models (both partial eta-squared) were calculated using the “*effectsize”* package in R (Navarro, 2015; Ben-Shachar et al., 2020). Further, we also investigated if there were any sex differences in the exploration and wayfinding success variables using two-factor ANOVAs.

##### Mediation analysis of group differences

In the second step, we investigated whether group differences in wayfinding success were mediated by group differences in any of the exploration variables, using causal mediation analysis models. Using the “mediation” R package (Tingley et al., 2014), a separate mediation model was run for each exploration variable exhibiting a significant group difference; in each model, age was the independent variable, wayfinding success was the dependent variable, and the respective exploration variables were the mediator variables.

##### Correlations between exploration and wayfinding, with PCA

In step three, we identified whether performance on the exploration variables was associated with wayfinding success in both the midlife and young groups respectively, by running separate ordinarily least square linear regression models for each of these groups. Due to the high multicollinearity and dimensionality exhibited amongst the exploration variables (see Supplementary Figure S1 for correlation matrix of exploration variables), we first conducted a principal components analysis (PCA) on the pooled midlife and young groups' dataset. Using PCA, we obtained new variables [i.e., principal components (PCs)] which represent a linear combination of all the exploration variables, and which are uncorrelated with one another. In addition to reducing the dimensionality of our dataset, this step importantly allows us to consider all the exploration variables in the downstream regression analyses.^2^

To run the PCA we used the “*PCATest”* R package (Camargo, 2022), which tests whether the derived PCs, as well as the exploration variables that load onto them, are significant using permutation-based statistical testing (at *p* < 0.05). Here, significant PCs denote axes representing a meaningful correlation of the respective exploration variables, and significant loadings denote meaningful contributions of the respective exploration variables to the PCs beyond random noise. The obtained significant PCs were used as the independent variables in separate regression models for the midlife and young groups, with wayfinding success as the dependent variables.

##### Classifier

In the fourth and final step, we explored whether we could classify participants into the young or midlife groups based on their exploration alone and on their wayfinding success alone, using binary logistic regression models. Here, the PCs we obtained from step three were used as the independent variables in a binary multiple logistic regression model, with group membership (young or midlife) as the dependent variable. We then ran a separate logistic regression model with wayfinding success as the independent variable and group membership (young or midlife) as the dependent variable. We conducted follow-up receiver operating characteristic (ROC) curves and computed the Area Under Curve (AUC) values to assess how well both logistic regression models could respectively classify the participants. Following this, a DeLong's test for paired ROC curves was performed using the “*pROC”* package in R (Robin et al., 2011), to assess statistical differences in the AUC values of both models.

## Results

### Group differences in exploration phase

**Figure 2:**
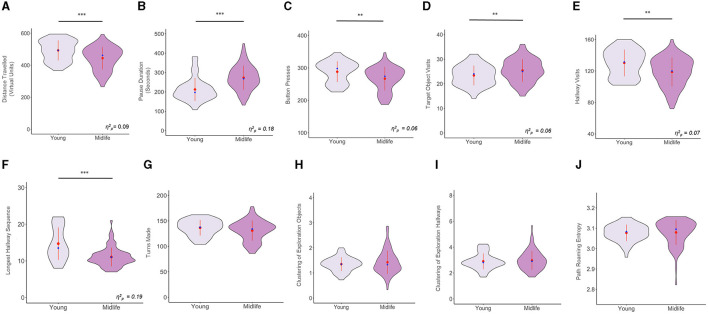
Violin plots showing group comparisons of all variables in the exploration phase, after controlling for sex. The y axes of all plots denote the raw values for all variables (including those that were transformed). The waves indicate the probability distribution of the variables, the red dots represent the group means, the red lines intersecting the red dots represent the group standard deviations, the blue dots represent the group medians, and the horizontal black lines indicate significant group differences. **(A)** Distance Traveled, **(B)** Pause Duration, **(C)** Number of Button Presses, **(D)** Number of Target Object Visits, **(E)** Number of Hallway Visits, **(F)** Longest Hallway Sequence, **(G)** Turns Made, **(H)** Clustering of Exploration of Objects, **(I)** Clustering of Exploration of Hallways, and **(J)** Path Roaming Entropy. **p < 0.01, ***p < 0.001.

Using sex as a covariate, we ran one-way ANCOVAs for all the exploration variables. We found that there were significant age effects for the following exploration variables after controlling for sex [*F*_(1, 134)_ = 13.59, *p* < 0.001 for distance traveled; *F*_(1, 134)_ = 30.23, *p* < 0.001 for pause duration; *F*_(1, 134)_ = 9.39, *p* = 0.002 for button presses; *F*_(1, 134)_ = 9.34, *p* = 0.002 for target object visits; *F*_(1, 134)_ = 10.73, *p* = 0.001 for hallway visits; *F*_(1, 134)_ = 31.82, *p* < 0.001 for longest hallway sequence). Meanwhile, the following variables did not exhibit significant age effects after controlling for sex [*F*_(1, 134)_ = 2.50, *p* = 0.115 for turns made; *F*_(1, 134)_ = 0.02, *p* = 0.870 for clustering of exploration of objects; *F*_(1, 134)_ = 0.09, *p* = 0.75 for clustering of exploration of hallways; *F*_(1, 134)_ = 0.61, *p* = 0.434 for path roaming entropy]. The results above are illustrated in Figure 2.

As an exploratory *post-hoc* analysis, we assessed how the exploration variables changed from block 1 to 2 of the exploration phase for the midlife and young groups respectively. These results are presented in detail in Table 1. In summary, we found differences between blocks in the young group for turns made, button presses, clustering of exploration of objects, and longest hallway sequence while a statistical trend was seen for path roaming entropy. Midlife adults had the same differences between blocks, with the exception of path roaming entropy which was not significant, and with the addition of target object visits. Notably, the longest hallway sequence got significantly longer for young adults, but shorter for midlife adults.

**Table 1:** Changes in exploration variables seen in midlife and young respectively from exploration phase block 1 to block 2.

**Group**	**Variable**	**Block 1 (mean; SD median)**	**Block 2 (mean; SD median)**	**p**	**Effect size (partial eta-squared)**
*Young*	Distance traveled (virtual units)	246.66 (31.00)	245.85 (40.04)	0.87	-
		246.62	249.75		
	Turns made	65.14 (8.81)	71.4 (9.22)	< 0.001	0.31
		66	72.5		
	Pause duration (seconds)	110.02 (23.16)	102.72 (42.84)	0.133	-
		109.03	90.76		
	Button presses	139.9 (15.42)	148.24 (19.68)	< 0.001	0.23
		139.5	154		
	Target object visits	11.88 (2.30)	11.50 (2.61)	0.34	-
		12	12		
	Hallway visits	63.88 (8.54)	66.34 (10.95)	0.08	-
		65	67.5		
	Clustering of exploration objects	0.82 (0.25)	0.99 (0.20)	< 0.001	0.23
		0.86	0.98		
	Clustering of exploration hallways	1.77 (0.51)	1.89 (0.43)	0.18	-
		1.68	1.85		
	Path roaming entropy	3.01 (0.11)	2.98 (0.08)	0.08	-
		3.03	3		
	Longest hallway sequence	11.48 (4.27)	13.14 (4.42)	0.04	0.08
		10	12.5		
*Midlife*	Distance traveled (virtual units)	224.13 (37.43)	219.76 (38.59)	0.14	-
		229.25	222.25		
	Turns made	63.31 (10.70)	67.59 (11.10)	< 0.001	0.18
		64	69		
	Pause duration (seconds)	139.79 (33.27)	133.79 (34.18)	0.03	0.05
		139.15	130.05		
	Button presses	130.73 (20.03)	136.08 (17.93)	< 0.001	0.16
		134	140		
	Target object visits	12 (2.60)	13.42 (2.76)	< 0.001	0.22
		12	13		
	Hallway visits	58.71 (10.09)	59.98 (9.89)	0.14	-
		60	61		
	Clustering of exploration objects	0.96 (0.31)	1.07 (0.34)	0.006	0.08
		0.92	1.01		
	Clustering of exploration hallways	1.93 (0.48)	2.03 (0.54)	0.13	-
		1.91	1.95		
	Path roaming entropy	2.96 (0.11)	2.97 (0.13)	0.6	-
		2.97	2.99		
	Longest hallway sequence	9.89 (2.31)	9.02 (2.36)	0.01	0.03
		10	9		

### Group differences in test phase

In the test phase, we first tested whether the midlife and young groups performed above chance level with regards to wayfinding success (where chance is 0.11 because there is a one in nine possibility of reaching the correct trial by chance on any trial) using one-tailed Wilcoxon Rank Sum tests (as both groups had a non-normal distribution). We found that the young group performed significantly above chance (W = 2,200, *p* < 0.001) while the midlife group overall performed close to or at chance (W = 4,089, *p* = 0.163). We next tested for group differences in wayfinding success, while controlling for sex. We found that compared to the young group, the midlife group had significantly less wayfinding success [*F*_(1, 134)_ = 6.35, *p* = 0.012].

**Figure 3:**
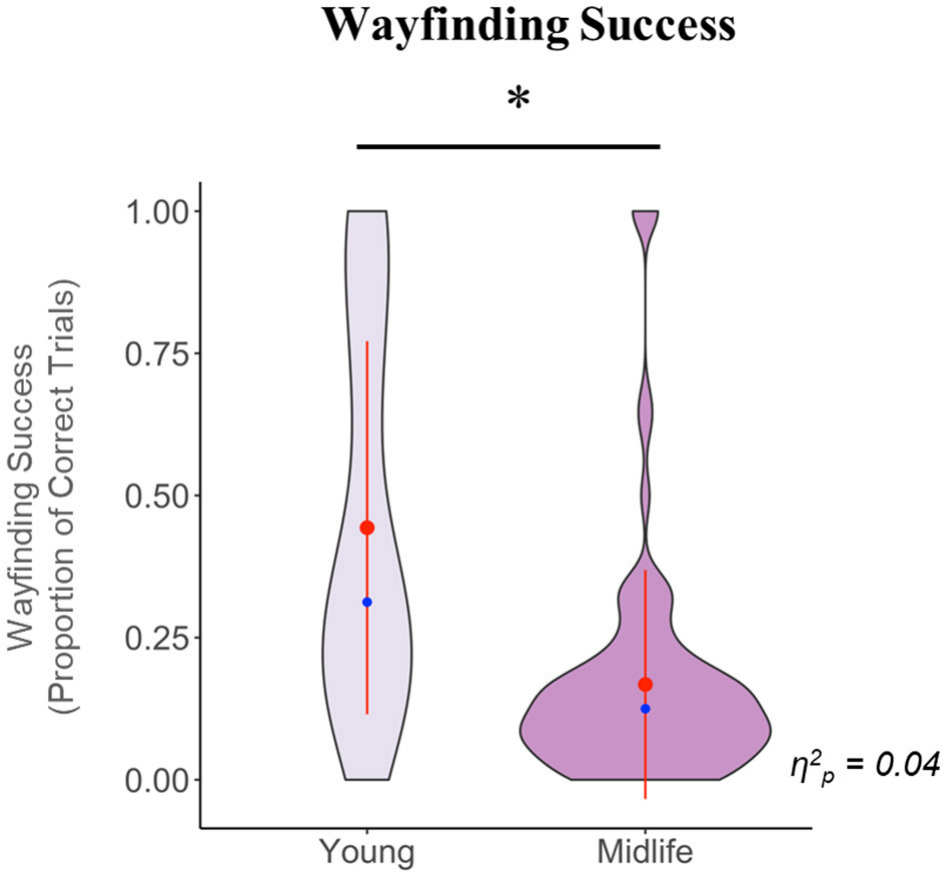
Violin plot showing the group comparison of wayfinding success in the test phase, after controlling for sex. The y axis denotes the raw, pre-transformed values for this variable. The waves indicate the probability distribution of the variables, the red dots represent the group means, the red lines intersecting the red dots represent the group standard deviations, the blue dots represent the group medians, and the horizontal black line indicates a significant group difference. Note that the standard deviation lines for the midlife group visually extend beyond the plot bounds; this is because due to the highly skewed distribution and presence of extreme values in this group, the standard deviation value is higher than the mean value. *p < 0.05.

The results for this phase are illustrated in Figure 3.

With sex differences being previously reported in exploration and navigation ability, we were interested to investigate such differences in our variables, as an exploratory *post-hoc* analysis. We found sex differences for many of the variables in both the exploration and test phases, and the full results of this analysis are presented in the Supplementary material.

### Mediation analysis of group differences

#### Wayfinding success

A total of six causal mediation models were run, one for each exploration variable that showed significant group differences (i.e., distance traveled, pause duration, button presses, target object visits, hallway visits, and longest hallway sequence). In each model, age (in years) was the independent variable, wayfinding success was the dependent variable, and each respective exploration variable exhibiting a significant group difference was the mediator variable. For each model, the direct effect (i.e., effect that age has on wayfinding success, after controlling for the mediator), average causal mediation effect (i.e., indirect effect that age has on wayfinding success, by acting through the mediator), and total effect (i.e., sum of the direct and mediation effect) are reported.

Our results showed that the group differences in distance traveled (direct effect = −0.007, *p* < 0.001; average causal mediation effect = −0.001, *p* = 0.04; total effect = −0.009, *p* < 0.001), target object visits (direct effect = −0.009, *p* < 0.001; average causal mediation effect = 0.0008, *p* = 0.03; total effect = −0.009, *p* < 0.001), and hallway visits (direct effect = −0.008, *p* < 0.001; average causal mediation effect = −0.0009, *p* = 0.05; total effect = −0.009; *p* < 0.001) all significantly partially mediated the group differences seen in wayfinding success.

These results highlight that the group differences in wayfinding success between the two age groups can partially be attributed to group differences in their exploration behavior. Specifically, it must be noted that an inconsistent mediation effect is seen for the target object visits variable, whereby the signs of the direct (−0.009) and mediation effects (0.0008) are in opposite directions (MacKinnon et al., 2007). Here, considering that the absolute magnitude of the direct effect is greater than the mediation effect, the direct effect is far stronger and over-rides the mediation effect. Meanwhile for the other two significant mediator variables (distance traveled and hallway visits), the signs of the direct effects and mediation effects are in the same direction and hence, these two effects complement one another.

#### Correlations between exploration and wayfinding with PCA

The PCA to derive PCs representing a linear combination of the exploration variables from the pooled midlife and young group datasets showed that PC1, PC2, and PC3 were significant and respectively accounted for 45.9%, 23.7%, and 13.2% of the total variance. After obtaining the significant PCs, we first identified which of the 10 variables significantly loaded onto each PC (see Table 2). From this list, we only considered variables with a loading score >|0.4|, in line with previous studies (Kumar et al., 2016; Iosa et al., 2022), to facilitate the interpretation of what the PCs represent.

**Table 2:** Variables significantly loading onto each principal component in pooled midlife and young dataset.

**Principal component**	**Significant variable loadings (name, magnitude)**
***PC1***	Distance traveled (0.435)
	Target object visits (0.347)
	Hallway visits (0.437)
	Turns made (0.346)
	Pause duration (−0.333)
	Clustering of exploration of hallways (0.210)
	Button presses (0.450)
***PC2***	Clustering of exploration of objects (−0.515)
	Clustering of exploration of hallways (−0.523)
	Path roaming entropy (0.621)
***PC3***	Longest hallway sequence (−0.784)

The variables significantly loading onto PC1 and passing the cut-off threshold were number of button presses (0.450), total hallway visits (0.437), and distance traveled (0.435). With these variables all measuring the amount of exploration one exhibits in the maze, we considered PC1 to represent *exploration quantity*. Based on these variables' loadings (all positive), higher PC1 scores represent higher exploration quantity (i.e., linear combination of greater button presses, higher number of hallway visits, and greater distance traveled). Variables significantly loading onto PC2 and passing the cut-off threshold were path roaming entropy (0.621), clustering of exploration of objects (−0.515), and clustering of exploration of hallways (−0.523). With these variables measuring how distributed or spread one's exploration was in the maze, we consider PC2 to represent *exploration quality*. Based on these variables' loadings, higher PC2 scores represent higher exploration quality (linear combination of higher path roaming entropy and less clustering of exploration of objects and hallways). The only variable significantly loading onto PC3 and passing the cut-off threshold was longest hallway sequence (−0.784). Here, we consider PC3 to represent *exploration of maze graph edges*, with higher PC3 scores representing less exploration of maze graph edges. To facilitate interpretation, we reversed all PC3 scores by multiplying them by −1; this results in higher PC3 scores representing greater exploration of maze graph edges.

**Figure 4:**
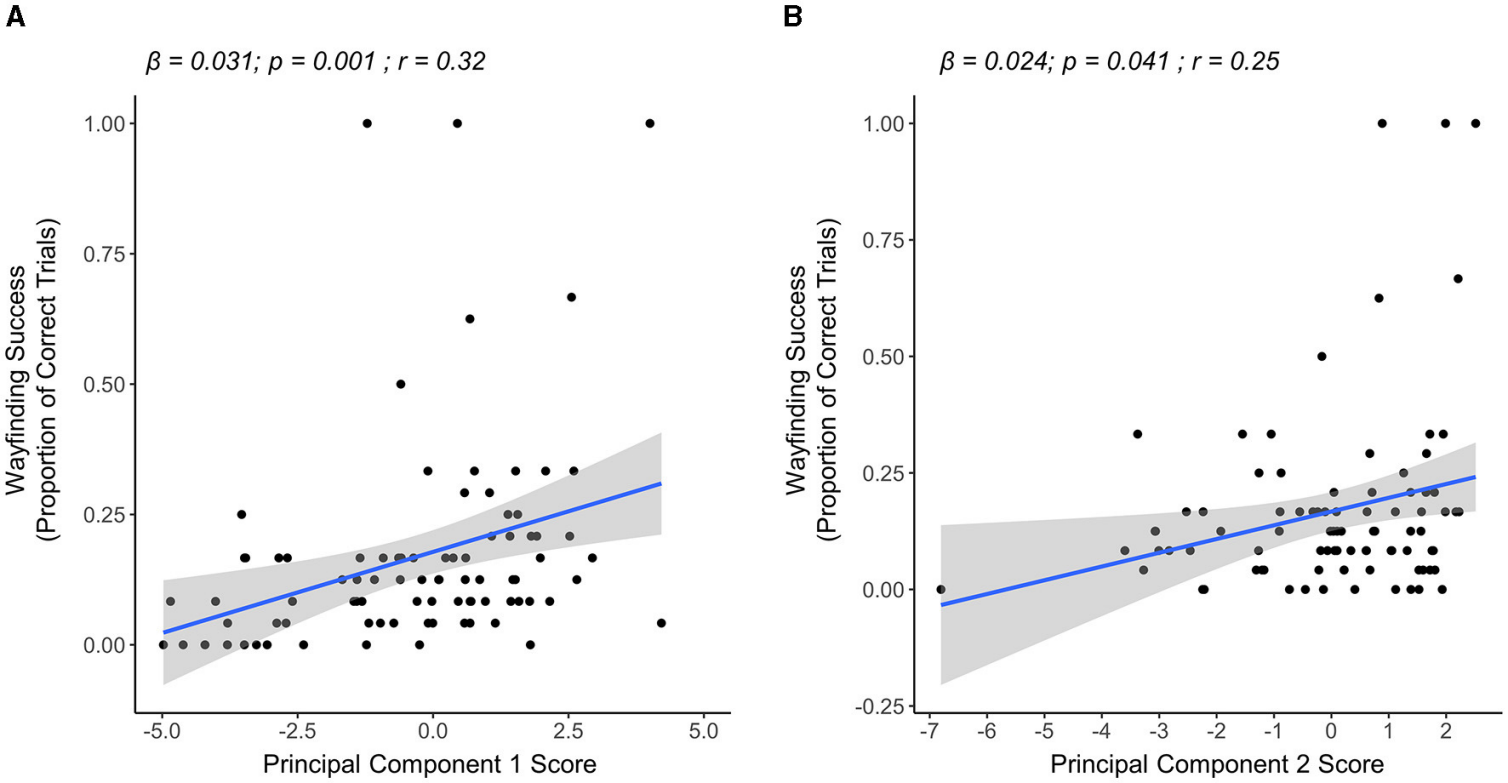
Linear regression plots showing the relationship between PCs and wayfinding success in the midlife group (*n* = 87). **(A)** The relationship between PC1 (exploration quantity) and wayfinding success. Higher PC1 scores were associated with higher wayfinding success. **(B)** Relationship between PC2 (exploration quality) and wayfinding success. Higher PC2 scores were associated with higher wayfinding success.

In the midlife group, the results of our multiple linear regression model showed that both PC1 and PC2 scores were significantly associated with wayfinding success in this group. Specifically, having higher PC1 scores (i.e., greater exploration quantity) (β = 0.031, *p* = 0.001) and higher PC2 scores (i.e., greater exploration quality) (β = 0.024, *p* = 0.041) were significantly associated with more wayfinding success (overall model R^2^ = 0.14, *p* < 0.001) (Figure 4). PC3 scores were not significantly associated with wayfinding success (β = 0.032, *p* = 0.249).

**Figure 5:**
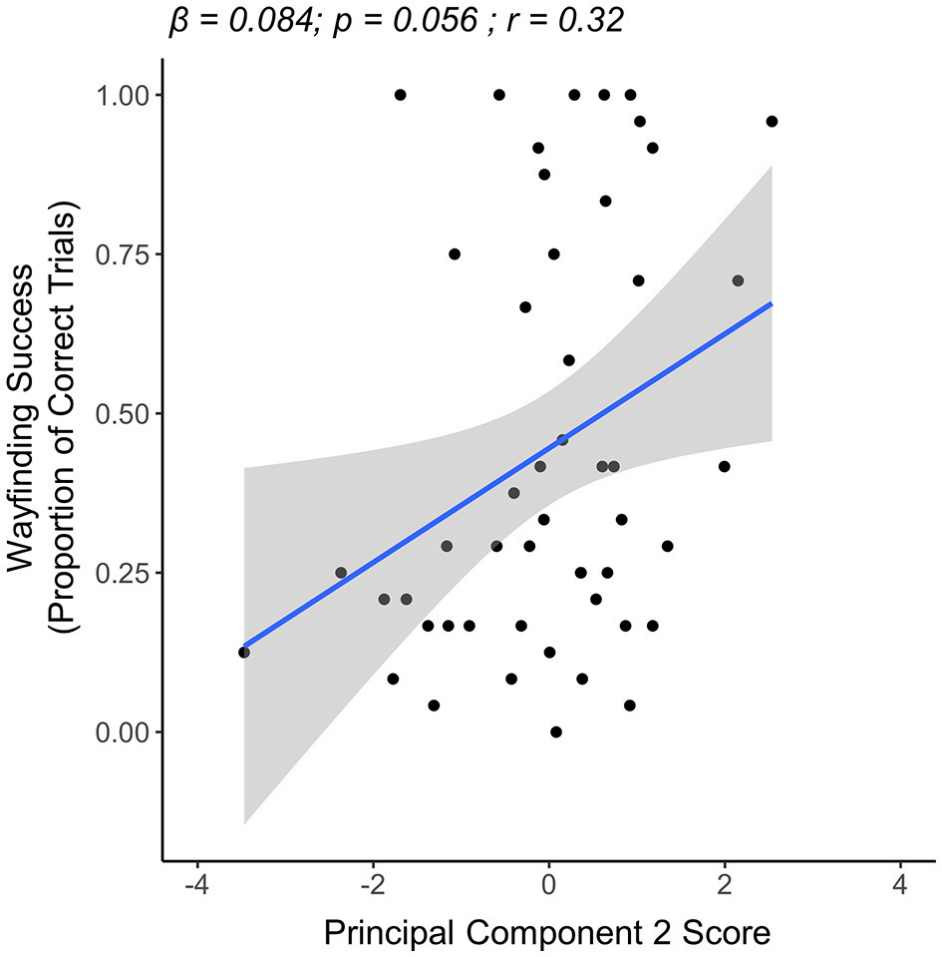
Linear regression plot showing the relationship between PC2 (exploration quality) and wayfinding success in the young group (n = 50). A statistical trend toward significance was seen for higher PC2 scores being associated with more wayfinding success.

In the young group, the results of our multiple linear regression model showed a trend for PC2 scores being associated with wayfinding success. Specifically, a trend was seen for having higher PC2 scores (i.e., higher exploration quality) being associated with more wayfinding success (β = 0.084, *p* = 0.056). Neither PC1 scores (i.e., exploration quantity) (β = −0.005, *p* = 0.815) nor PC3 scores (i.e., exploration of maze graph edges) (β = −0.012, *p* = 0.779) were significantly associated with wayfinding success. Here, the overall model relationship was not significant (overall model R^2^ = 0.046, *p* = 0.162) (Figure 5).

As a *post-hoc* sensitivity analysis, we ran the PCA analysis on the midlife and young group datasets separately. In both age groups, we obtained similar multiple regression model results to the main analysis (see Supplementary material for details).

#### Classifier

Here, we explored whether we could classify our participants into the young or midlife groups based on their exploration PCs and wayfinding success variables respectively.

**Figure 6:**
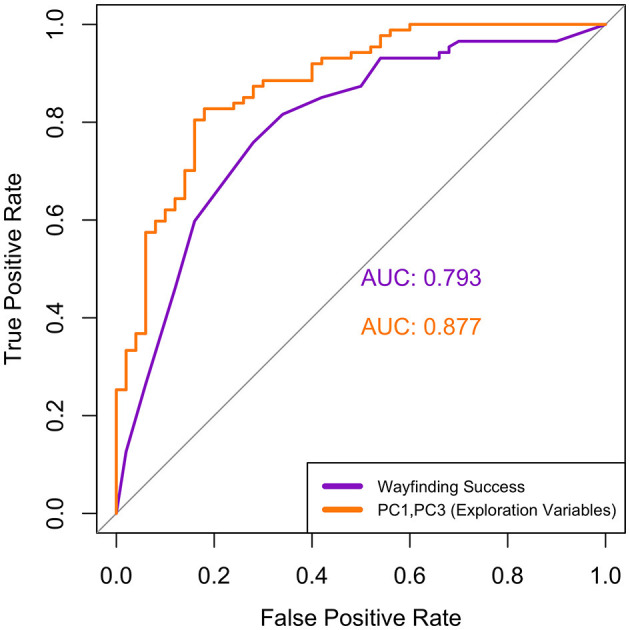
Comparison of ROC curves for the logistic regression models with wayfinding success (purple, AUC = 0.793) and a combination of the PCs (orange, AUC = 0.877) as the independent variables. Although the combined exploration model has a higher AUC, the two curves were not significantly different (*p* = 0.107).

Using PC1, PC2, and PC3 from the pooled midlife and young dataset as multiple independent variables in our first logistic regression model, we found that only PC1 and PC3 scores significantly predicted group membership. Specifically, higher PC1 scores (i.e., greater exploration quantity) significantly decreased odds of being classified into the midlife group (odds ratio = 0.678, *p* = 0.002); likewise, higher PC3 scores (i.e., greater exploration of maze graph edges) significantly decreased odds of being classified into the midlife group (odds ratio = 0.18, *p* < 0.001). PC2 scores (i.e., denoting exploration quality) did not significantly predict group membership (odds ratio = 0.99, *p* = 0.965). The follow-up ROC curve for the combined model with only PC1 and PC3 as predictors had an AUC value of 0.877 (Figure 6), which is considered to represent an acceptable discriminatory ability (Hosmer and Lemeshow, 2000).

For our second logistic regression model, with wayfinding success as the independent variable and group (young or midlife) as the dependent variable, the results showed that an increase in the wayfinding success score significantly decreased the odds of being classified into the midlife group (odds ratio = 0.020, *p* < 0.001). The follow-up ROC curve for this model had an AUC value of 0.793 (Figure 6), which is considered to represent an acceptable discriminatory ability (Hosmer and Lemeshow, 2000).

Although the ROC curve for the model with exploration PCs as predictors had a numerically higher classification score than that of wayfinding success alone, a DeLong's test for two correlated ROC curves showed that there were no significant differences between the AUC values of the wayfinding success curve and the PC1 + PC3 ROC curve (*p* = 0.107).

## Discussion

In this study, we identified differences between spatial exploration behavior in midlife and young adults, and highlighted how certain aspects of this behavior are associated with spatial memory in the midlife age group. Relative to young adults, midlife adults differ in the way they actively explore a novel spatial environment as well as their ability to navigate to specific locations within this environment using their spatial memory. In particular, group differences in two exploration variables (*distance traveled* and *hallway visits*) partially mediated the group differences seen in spatial memory performance. Furthermore, both exploration quantity and quality were associated with spatial memory in the midlife group, whereas a trend was seen for only exploration quality being associated with spatial memory in the young group. Finally, measures of spatial exploration behavior and spatial memory were equally able to classify our participants into the midlife and young groups.

### Differences between midlife and younger adult exploration behavior and subsequent knowledge

Firstly, our results from the test phase show that midlife adults exhibited poorer wayfinding success when compared to young adults, likely reflecting age-related declines in spatial memory. In addition to being in line with the well-reported deficits to spatial memory in older adults (Lithfous et al., 2013; Lester et al., 2017), this finding also adds to the growing body of evidence showing such deficits in the midlife population (Ritchie et al., 2018; Williams et al., 2019; Yu et al., 2021). Our results from the exploration phase show that the midlife group explored the maze environment in a different manner than the young group, with regards to the variables of distance traveled, pause duration, button presses, target object visits, hallway visits, and longest hallway sequence. Specifically, our findings of the midlife group having less distance traveled, longer pause durations, and fewer button presses reflect upon the midlife group exhibiting overall less exploration in the maze.

This finding complements the animal literature, in which studies have reported less exploration behavior with age, with aged rodents and zebrafish exhibiting a lower tendency to explore previously unexplored arms of a maze (Willig et al., 1987; Lamberty and Gower, 1990; Lalonde, 2002; Yu et al., 2006) and some aged wasps exhibiting less patch-searching behavior in an experimental environment (Thiel et al., 2006). Although the results of these studies suggest less exploration in the form of less coverage of an environment, and our results indicate less exploration in the form of less quantity of exploration, all findings taken together indicate less exploratory behavior of spatial environments for aging across species.

Our second set of findings from the exploration variables offers insight into group differences in what specific features of the maze environment were explored. The midlife group made fewer hallway visits and had shorter longest hallway sequences compared to the young, in line with our findings of less exploration. However, they actually had more target object visits than the young adults, which provided them with more exposure to their eventual targets in the test phase. These findings suggest that the midlife group prioritized learning specific locations within the environment (i.e., the target object locations), whereas the young group seemed to prioritize learning the connections between different locations (i.e., hallways) and the overall layout of the maze (i.e., hallway sequences). Shedding further light on this are our *post-hoc* findings, which reveal that from block 1 to 2 of the exploration phase, the midlife group increased their target object visits and decreased their longest hallway sequences while the young group increased their longest hallway sequences. Indeed, these findings give further credence to the notion that these exploration patterns reflect specific learning styles of the maze environment seen in the two age groups, as opposed to purely random exploration. To the best of our knowledge, this is the first finding of changes to spatial exploration behavior in healthy midlife adults (i.e., reduced overall exploration & biases in features of the environment being explored).

This pattern of results is in line with findings from a recent study assessing the visuospatial encoding patterns of younger and older adults as they studied and learned the spatial configuration of a cluster of objects in a virtual room (Segen et al., 2022). Here, younger adults focused more on learning the object cluster layouts by visually studying the spatial relationships between the objects, while the older adults focused more on learning the objects' locations in relation to external landmarks (i.e., paintings) in the environment. These results taken together with our findings suggest an age-related shift from learning overall spatial layouts toward a focus on specific features of the environment, such as the objects themselves.

Several potential mechanisms may underlie the changes in exploration behavior that we see in our midlife group, some of which may in turn also offer insight into reasons underlying their deficits in wayfinding success. From a behavioral perspective, these changes can be discussed in the context of the exploration vs. exploitation trade off—that is, whether one favors exploring new environments and learning more about them, or favors making use of known aspects of the current environment (Sutton and Barto, 1998). Previous studies have shown that with increasing age, a shift occurs in the balance of this tradeoff from exploration toward exploitation (Eliassen et al., 2007; Wang et al., 2009). One possible reason for this age-related shift may be a functionally adaptive or compensatory response. Specifically, midlife adults likely possess fewer cognitive resources (Dohm-Hansen et al., 2024), and in situations requiring the use of their cognitive abilities, they look to minimize the expenditure of these resources. It is possible that the exploration patterns of the midlife group reflect them choosing the relatively simpler strategy of learning where the target objects are located, as opposed to the more cognitively demanding strategy of learning the overall maze layout (as observed in the young adults) (Varner et al., 2016). Indeed, this line of reasoning is consistent with age-related compensatory shifts seen in the usage of an allocentric to an egocentric reference frame during navigation (Rodgers et al., 2012), which are already evident by midlife (Yu et al., 2021); specifically, we speculate that the midlife group could predominantly be utilizing an egocentric strategy to learn the environment by attempting to learn a series of turns to get from one object to the next. Meanwhile the young group, who likely possess more cognitive resources, may choose to utilize the more optimal and demanding allocentric strategy to learn the environment layout, as evidenced by their greater hallway visits and longer hallway sequences (Varner et al., 2016). In addition to shifts in spatial reference frames, the midlife group may be less facile with computer controls compared to the young group. Although the maze used relatively simple button presses, unfamiliarity with the controller or lack of video gaming experience might also underlie their use of the relatively simpler target object-focused exploration strategy, as more of their cognitive resources could be taken up by the act of moving through the maze. Overall, the midlife group's exploration behavior could explain their poorer wayfinding success in the test phase, as their possible strategy of learning turns from one target object to another could have proven impractical with all the target objects being transformed into red spheres during this phase.

Other studies offer insight into the potential neural mechanisms that may explain why aging is associated with less exploration and impaired wayfinding success. Specifically, aging has been shown to be associated with deficits to the modulation of neural activity by catecholamines (i.e., dopamine and norepinephrine), which may already manifest in midlife (Twarkowski and Manahan-Vaughan, 2016). With catecholamines being suggested to modulate novelty seeking and exploration behavior (Cohen et al., 2007; Eppinger et al., 2011; Mata et al., 2013), age-related deficits in catecholaminergic neuromodulation could result in less exploratory behavior. Furthermore, a recent study has suggested theta oscillations (i.e., synchronous neuronal firing activity in the theta frequency) in the prefrontal and parietal regions as playing an important role for learning environments through active exploration in younger adults (Chrastil et al., 2022). Considering findings that older adults exhibit reduced theta oscillations in the left frontal regions during spatial encoding (Lithfous et al., 2015), it is possible that such reductions may already be seen in midlife, potentially underlying the lesser exploration seen in this group when learning the maze environment, however this notion requires further investigation. Lastly, a functional MRI study of free exploration and learning in a virtual environment showed reduced activity during exploration in older adults compared to younger adults throughout the navigation network, specifically in the medial temporal (i.e., posterior hippocampus, parahippocampal gyrus) and parietal lobes (i.e., retrosplenial cortex and other parietal regions) (Moffat et al., 2006). Although it is unclear whether such reduced activity in the navigation network may already be seen in midlife, it is possible that this may also represent an underlying factor associated with the lesser exploration seen in our midlife adults. Moreover, with regions of the navigation network being reported to underlie spatial memory, notable structural changes that occur to these regions in midlife, especially in the medial temporal lobe, could also explain the deficits to wayfinding success seen in the midlife group (Fjell et al., 2013; Hasan et al., 2016).

### Relationship between spatial exploration and wayfinding success (midlife and young)

Our linear regression model results show that different aspects of exploration were important for wayfinding success performance in the midlife and young, respectively. In the midlife group, both exploration quantity and quality were important for wayfinding success, while in the young, a trend was seen for only exploration quality being important for wayfinding success. Findings from previous studies investigating exploration behavior in young adults have reported an association between specific exploration quantity variables (as mentioned in the Introduction) and more navigation success (i.e., acquisition of route and survey knowledge) (Gagnon et al., 2018; Munion et al., 2019). Our findings add to these studies by showing that aspects of exploration are associated with navigation success, with respect to acquiring and using spatial graph knowledge. In addition, we also report for the first time the relationship between spatial exploration behavior and spatial memory in the midlife population.

The exact underlying reasons as to why greater exploration quantity seems to be beneficial for wayfinding success in the midlife, and not in the young, is at present unclear. With the maze environment having finite boundaries, it can be reasoned that having more exploration in the maze leads to individuals being exposed more to/repeatedly encountering certain locations throughout the maze. In fact, such repeated exposure could be beneficial specifically for the midlife group by aiding their learning of the maze environment, in line with results from a previous study showing how backtracking in a novel environment may improve spatial memory for that environment, albeit in young adults (Mukawa et al., 2015). Alternatively, we speculate that regardless of age, there may be a specific exploration quantity threshold that once crossed, is beneficial for spatial memory. The young group explored the maze more in general, so they may have well crossed this threshold while only some of the midlife group did which might explain the specific relationship between exploration quantity and wayfinding success observed in this group. Overall, our results across both age groups suggest an important role for spatial exploration for subsequent spatial memory.

Results from our mediation analysis shed further light into the association between spatial exploration and spatial memory. Specifically, we showed that the group differences in wayfinding success were partially due to the midlife group traveling less distance and making fewer hallway visits in the maze when compared to the young. Although this finding implies that the wayfinding performance of the midlife group could have been improved partially through the modification of their exploration behavior, this remains to be investigated by future studies.

Lastly, when assessing the relationship between spatial exploration and spatial memory, it is worth acknowledging that the two may be intrinsically related. Specifically, in the exploration phase the participants are actively forming spatial memories of the maze locations and layout, and these memories may in turn bias their exploration behavior in the maze (i.e., those with poorer spatial memory exhibiting a different exploration pattern vs. those with stronger spatial memory). In the current study, the extent to which such intrinsic associations between the two contributed to the relationships observed in our multiple regression models are at present unclear. Indeed, future studies should attempt to elucidate this intrinsic association further by investigating how individual differences in baseline spatial memory abilities impact spatial exploration, and by identifying potential differences in the underlying neural pathways for both behaviors.

### Classification of age groups from spatial exploration and wayfinding success

Our results from the classification analysis showed that the exploration and wayfinding success variables were able to classify participants into the midlife group to a similar degree, implying both behaviors to represent equally sensitive markers for cognitive aging. Of note, the aspects of exploration that could significantly predict group membership were exploration quantity and exploration of maze graph edge structure. This finding suggests that in terms of spatial exploration behavior, the defining features which distinguish the midlife from the young are reductions in overall exploration and exploration of maze graph edge structure. With the medial temporal lobe structures being implicated in both spatial exploration and spatial memory (Moffat et al., 2006; Konishi and Bohbot, 2013), age-related alterations to these structures seen in midlife (Fjell et al., 2013; Hasan et al., 2016) could result in changes to exploration and spatial memory appearing at a similar time in the aging process, potentially explaining the similar sensitivity of both behaviors in being markers for cognitive aging.

### Study implications

Overall, the novel findings of our study have important wider implications. By shedding light on how spatial exploration behavior changes with age, our findings first and foremost increase our overall theoretical understanding of the impact that age has on different aspects of spatial navigation. From a clinical perspective, recent findings have linked poorer baseline navigation ability with future incidence and clinical progression of mild cognitive impairment and AD (Wood et al., 2016; Verghese et al., 2017; Levine et al., 2020). Thus, improving navigation abilities in midlife adults, potentially through training aspects of exploration quantity and quality, could help delay the onset of these clinical conditions in addition to helping enhance their real-world functioning. Lastly, although we found spatial exploration to be a similarly sensitive marker for cognitive aging to spatial memory, the novelty of this finding lies in highlighting exploration behavior as being a component of navigation that is affected by the aging process. These results motivate future studies to investigate whether additional alterations to this behavior can be seen in pathological aging, such as AD. Such future investigations can elucidate whether potential alterations to exploration behavior can be considered a novel cognitive marker for the disease, in addition to the currently reported spatial memory (Kunz et al., 2015; Allison et al., 2016; Coughlan et al., 2019).

### Limitations

Despite our novel findings, there are some limitations to our study that need to be addressed. Firstly, the relatively poor performance of the midlife group on the test phase suggests they had difficulties in learning the maze environment, which is a point worth considering when assessing the impact of exploration behavior on subsequent spatial memory in this group. However, our focus here was on how midlife adults explore, which clearly differed from young adults. The mediation analysis suggests that these differences in exploration might partially underlie the poorer performance in midlife adults.

More generally, our experimental setup involved participants being stationary and navigating on a desktop VR. This setup lacks the involvement of self-motion cues or locomotion, both of which are critical for navigation (Diersch and Wolbers, 2019). Thus, the extent to which our results capture group differences that may be seen in more ecological settings (i.e., navigating by walking) is at present unclear. Indeed, validation of our findings by future studies using immersive VR environments, which incorporate walking, is warranted. This would be especially relevant considering recent findings suggesting that age-related differences in spatial memory seen in desktop VR environments are attenuated in ambulatory VR environments (Hill et al., 2023).

The extent to which group differences in cognitive factors, personality, or life experiences may have impacted the group differences seen in the exploration variables is at present unclear. We did not collect any background information on participants' video gaming experience or explicitly measure their visuospatial working memory abilities, both of which are factors that have been suggested to influence the acquisition of spatial knowledge in virtual and real-world environments (Richardson et al., 2011; Muffato et al., 2022). In addition, we did not consider the potential role that curiosity may have played in underlying our results, in light of findings from a recent study suggesting that curiosity levels impact the magnitude of path roaming entropy during exploration (Cen et al., 2022). Lastly, our measure of wayfinding success predominantly tests participants' spatial graph knowledge (Chrastil and Warren, 2014), and we did not make separate assessments of participants' route or survey knowledge. An interesting direction for future work could be to investigate how different patterns of exploration may impact the formation of these other types of spatial representations, and how this may differ between the young and midlife.

## Conclusions

In conclusion, the results of our study provide novel insight into the impact that normal aging may have on spatial exploration behavior and how different aspects of exploration benefit spatial memory in the midlife and young. We show that differences in spatial exploration behavior occur alongside differences in spatial memory in the midlife compared to young adults, suggesting that both behaviors are similarly sensitive to age effects. Knowledge generated from our findings can potentially contribute to the development of training interventions that can help midlife adults improve their navigation abilities, as well as provide a strong platform for future studies to investigate potential changes in spatial exploration behavior as representing an early cognitive marker for AD.

## Data availability statement

The datasets presented in this study can be found in online repositories. The names of the repository/repositories and accession number(s) can be found below: the anonymized study data of this manuscript can be found on OSF (https://osf.io/eskza/?view_only=909acb80078643a59d6ca4af68746409). The R code used to analyze the data in this manuscript can be found on Github (https://github.com/Vaisakh94/Midlife-Exploration-Manuscript.git).

## Ethics statement

The studies involving humans were approved by the University of California, Santa Barbara Human Subjects Committee. The studies were conducted in accordance with the local legislation and institutional requirements. The participants provided their written informed consent to participate in this study.

## Author contributions

VP: Conceptualization, Formal analysis, Investigation, Methodology, Project administration, Visualization, Writing – original draft, Writing – review & editing. DC: Formal analysis, Investigation, Methodology, Writing – review & editing. SY: Data curation, Project administration, Writing – review & editing. FR: Formal analysis, Writing – review & editing. MH: Funding acquisition, Project administration, Resources, Supervision, Writing – review & editing. EJ: Funding acquisition, Project administration, Resources, Supervision, Writing – review & editing. EC: Conceptualization, Funding acquisition, Investigation, Methodology, Project administration, Resources, Supervision, Writing – review & editing.

## Footnotes

^1^American National Adult Reading Test.

^2^Our rationale for running the PCA on the pooled dataset, as opposed to in the midlife and young datasets separately, was to obtain the same set of PCs for both groups. This will subsequently enable us to make a direct comparison of the results of the regression models run in the two groups when assessing how exploration behavior is related to wayfinding success.
