# Supplementary material for: Less spatial exploration is associated with poorer spatial memory in midlife adults
Source: Front Aging Neurosci. 2024 Jun 11;16:1382801. doi: 10.3389/fnagi.2024.1382801 (PMC11196421; doi:10.3389/fnagi.2024.1382801)
Supplement: Supplementary file 1 [file Data_Sheet_1.DOCX]

***Supplementary Material***

# **Less spatial exploration is associated with poorer spatial memory in midlife adults**

**Vaisakh Puthusseryppady, Daniela Cossio, Shuying Yu, Farnaz Rezwana, Mary Hegarty*, Emily G. Jacobs*, Elizabeth R. Chrastil***

***Correspondence:** Prof. Mary Hegarty ([hegarty@ucsb.edu](mailto:hegarty@ucsb.edu)), Prof. Emily G. Jacobs ([emily.jacobs@psych.ucsb.edu](mailto:emily.jacobs@psych.ucsb.edu)), Prof. Elizabeth R. Chrastil ([chrastil@uci.edu](mailto:chrastil@uci.edu))


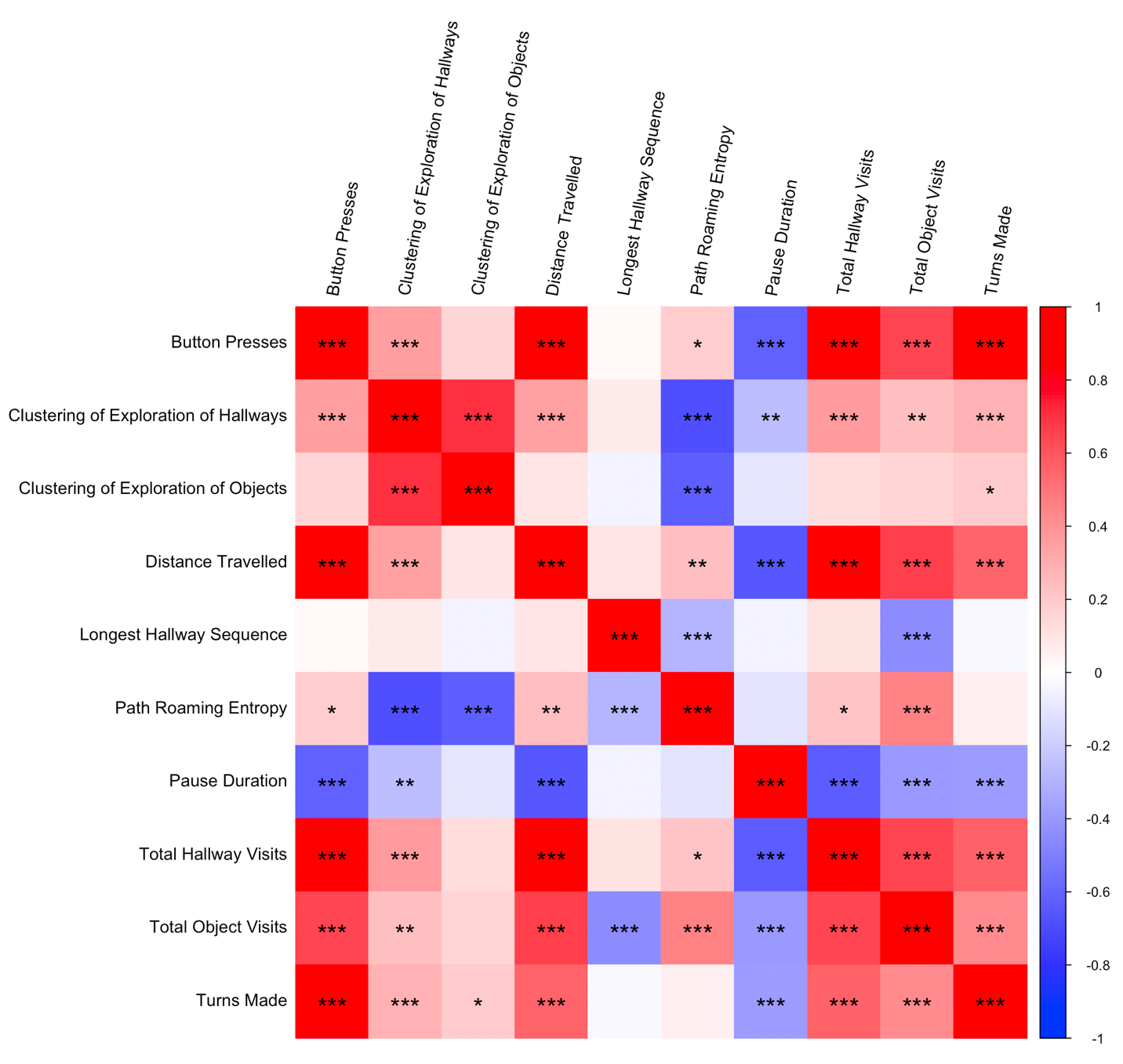


**Supplementary Figure** **1**: Correlogram illustrating all Pearson’s correlations of the exploration variables in the pooled midlife and young dataset. All correlations are colored based on the size and direction of the correlation coefficient values. ***p < 0.001, ** p < 0.01, * p < 0.05.

**Sex Differences in the Exploration and Test Phases**

Two-factor ANOVAs (sex: male, female; age: young, midlife) were run for each variable in the exploration and test phases, with age and sex as the independent variables and the respective exploration/test phase variables as the dependent variable. Significant interactions (if any) were followed up with Tukey’s HSD post-hoc tests, which were corrected for multiple comparisons.

Our results showed significant main effects of sex (in addition to age) on the following exploration phase variables (note: only main effects of sex are reported as age effects mirror results already reported above) – compared to females, males had greater distance travelled (F (1,133) = 6.935, p = 0.009, η^2^ = 0.020; males mean = 482.64, females mean = 450.67), made more button presses (F(1,133) = 4.586, p = 0.034, η^2^ = 0.013; males mean = 283.34, females mean = 270.03), had shorter pause durations (F(1,133) = 4.477, p = 0.036, η^2^ = 0.004; males mean = 236.19, females mean = 259.32) and had more hallway visits (F(1,133) = 5.513, p = 0.020, η^2^= 0.016; males mean = 127.83, females mean = 120.33) . No age × sex interaction was seen in any of these variables, except for pause duration (F(1,133) = 4.475, p = 0.036, η^2^ = 0.026). Here, post-hoc pairwise comparisons showed that midlife females had longer pause durations than young males and females (p < 0.001 for both) while midlife males had longer pause durations than young females (p = 0.039).

We also found a statistical trend for the effect of sex (but not age) for clustering of exploration of objects. Specifically, males exhibited a trend for less clustered exploration of objects compared to females (F(1,133) = 2.837, p = 0.094, η^2^=0.016; males mean = 1.31, females mean = 1.44). Lastly, although no significant effects of sex nor age was found for clustering of exploration of hallways, a significant age × sex interaction was found (F(1,133) = 4.353, p = 0.038, η^2^= 0.031). However, none of the post-hoc pairwise comparisons were shown to be significant.

In the test phase, there was a significant main effect of sex (in addition to age) in wayfinding success (F(1,133) = 66.44, p < 0.001, η^2^ = 0.18), with males having greater wayfinding success than females (males mean = 0.47, females mean = 0.16). There was a significant age × sex interaction (F(1,133) =16.91, p < 0.001, η^2^ = 0.067), with significant sex differences being seen in the young (p < 0.001) and only a statistical trend seen in the midlife participants (p = 0.079). In addition, young males outperformed midlife females on wayfinding success (p < 0.001).

**Correlations Between Exploration and Wayfinding, with PCA**

***Midlife***

The PCA for the midlife group dataset showed that PC1 and PC2 were significant and respectively accounted for 47.5% and 24.9% of the total variation in the dataset. After obtaining the significant PCs, we first identified which of the 10 variables significantly loaded onto each PC (see Table S1). From this list, we only considered variables with a loading score > |0.4|, in line with previous studies (Iosa et al., 2022; Kumar et al., 2016), to facilitate the interpretation of what the PCs represent.

Supplementary Table 1: Variables significantly loading onto each principal component in midlife

| Principal Component | Significant Variable Loadings  (Name, Magnitude) |
| --- | --- |
| *PC1* | Distance Travelled (−0.425) |
|  | Total Object Visits (−0.409) |
|  | Total Hallway Visits (−0.428) |
|  | Turns Made (−0.322) |
|  | Pause Duration (0.332) |
|  | Clustering of Exploration of Hallways (−0.192) |
|  | Button Presses (−0.438) |
| *PC2* | Clustering of Exploration of Objects (−0.563) |
|  | Clustering of Exploration of Hallways (−0.537) |
|  | Path Roaming Entropy (0.592) |

The variables which significantly loaded onto PC1 and passed the cut-off threshold were button presses (−0.438), total hallway visits (− 0.428), distance travelled (−0.425), and total object visits (−0.409). With these variables all measuring the amount of exploration one exhibits in the maze, we considered PC1 to represent *exploration quantity*. Based on these variables’ loadings (all negative), higher PC1 scores represent lower exploration quantity (i.e., linear combination of fewer button presses, fewer object and hallway visits, and less distance travelled). To make the PC1 scores more intuitive with regards to what they represent and for easier comparison across groups, we reversed all the scores by multiplying them by −1; this results in higher PC1 scores now representing higher exploration quantity.

The variables significantly loading onto PC2 and passing the cut-off threshold were clustering of exploration of objects (−0.563), clustering of exploration of hallways (−0.537), and path roaming entropy (0.592). With these variables measuring how distributed or spread one’s exploration was in the maze, we consider PC2 to represent *exploration quality*. Based on these variables’ loadings, higher PC2 scores represent higher exploration quality (i.e., linear combination of lower clustering of exploration values and higher path roaming entropy values).

The results of our multiple linear regression model showed that both PC1 and PC2 scores were significantly associated with wayfinding success in the midlife group. Specifically, having higher PC1 scores (i.e., greater exploration quantity) (β = 0.031, p < 0.001) and higher PC2 scores (i.e., greater exploration quality) (β = 0.027, p = 0.036) were significantly associated with higher wayfinding success (overall model R^2^ = 0.14, p < 0.001)(Fig. S2).


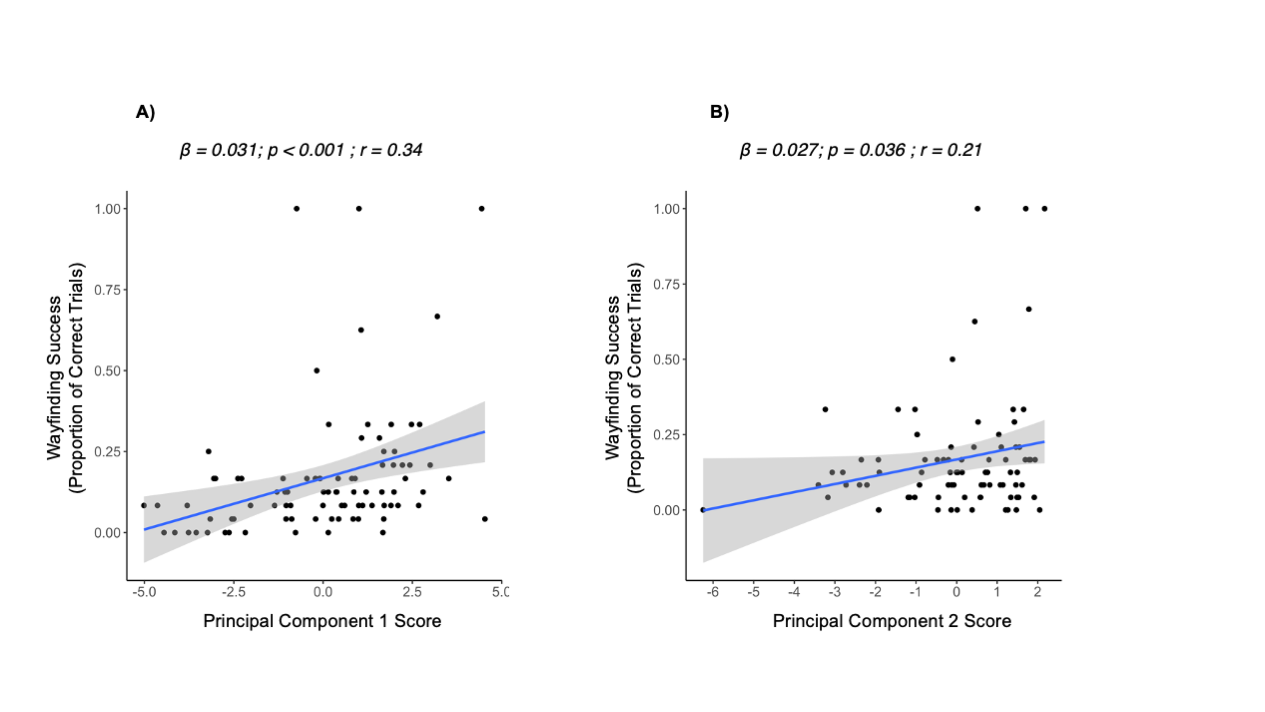


**Supplementary Figure** **2**: Linear regression plots showing the relationship between PCs and wayfinding success in the midlife group. (A) The relationship between PC1 (exploration quantity) and wayfinding success. Higher PC1 scores were associated with higher wayfinding success. (B) Relationship between PC2 (exploration quality) and wayfinding success. Higher PC2 scores were associated with higher wayfinding success.

***Young***

The PCA for the young group dataset showed that PC1 and PC2 were significant and respectively accounted for 50.3% and 23.3% of the variation in the dataset. Similar to the midlife, we identified which of the exploration variables significantly loaded onto each PC (see Table S2).

Supplementary Table 2: Variables significantly loading onto each principal component in young

| Principal Component | Significant Variable Loadings  (Name, Magnitude) |
| --- | --- |
| *PC1* | Distance Travelled (0.418) |
|  | Total Object Visits (0.339) |
|  | Total Hallway Visits (0.410) |
|  | Turns Made (0.351) |
|  | Pause Duration (−0.295) |
|  | Clustering of Exploration of Objects (0.265) |
|  | Clustering of Exploration of Hallways (0.234) |
|  | Button Press (0.430) |
| *PC2* | Clustering of Exploration of Hallways (0.488) |
|  | Path Roaming Entropy (−0.633) |
|  | Longest Hallway Sequence (0.417) |

The variables significantly loading onto PC1 and passing the cut-off threshold were number of button presses (0.430), distance travelled (0.418), and total hallway visits (0.410). Based on these variables, which replicate those seen in the midlife group, we consider PC1 to represent *exploration* *quantity*, with higher PC1 scores indicating higher exploration quantity (i.e., combination of greater button presses, greater distance travelled, and more hallway visits). The variables significantly loading onto PC2 and passing the cut-off threshold were path roaming entropy (−0.630), clustering of exploration hallways (0.480), and longest hallway sequences (0.410). As these variables all measure distribution/spread of one’s exploration, we consider PC2 to represent *exploration quality.* Based on the variables’ loadings here, higher PC2 scores represents lower exploration quality (i.e., linear combination of lower path roaming entropy, higher clustering of exploration of hallways, and longer hallway sequences). To make the PC2 scores more intuitive, we reversed all scores by multiplying them by − 1; this results in higher PC2 scores now representing higher exploration quality.

The results of our multiple linear regression model showed that only PC2 scores were significantly associated with wayfinding success in the young group. Specifically, having higher PC2 scores (i.e., higher exploration quality) was significantly associated with higher wayfinding success (β = 0.069, p = 0.024), while PC1 scores (i.e., exploration quantity) were not significantly associated with wayfinding success (β = − 0.006, p = 0.748). However, it must be noted that the significance of the overall model relationship was marginal (overall model R^2^ = 0.067, p = 0.072)(Fig. S3).


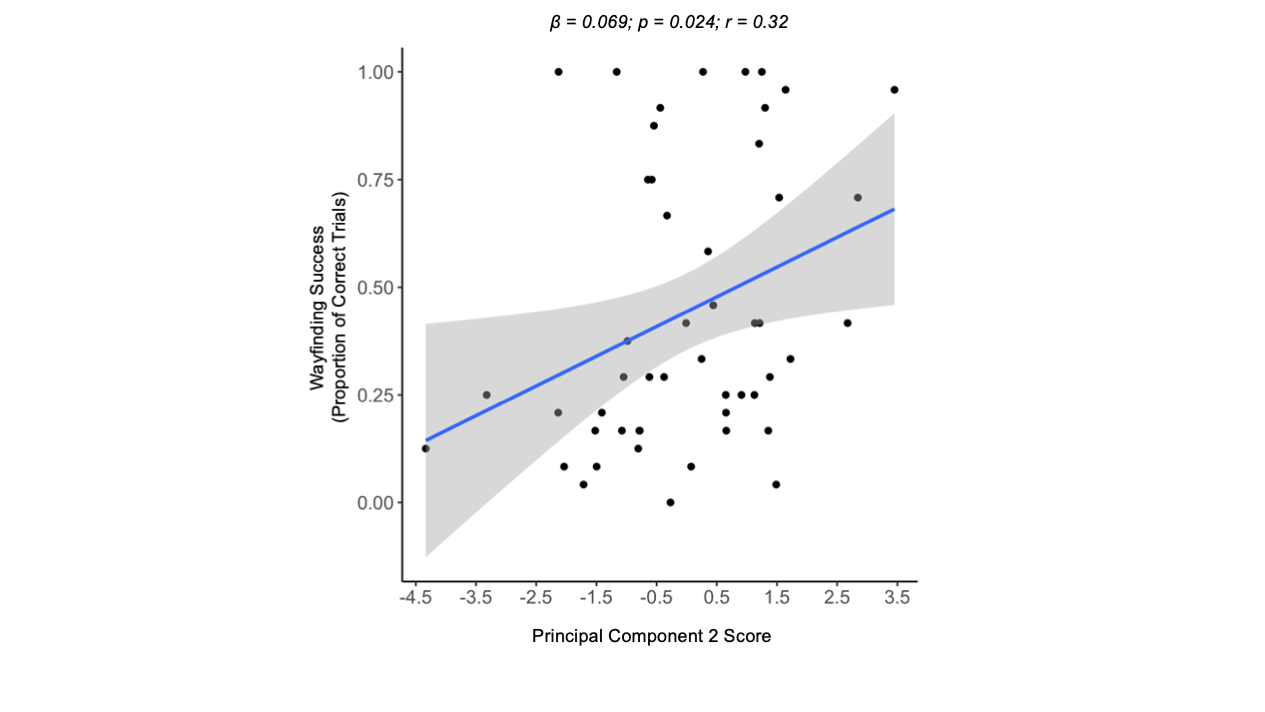


**Supplementary Figure** **3**: Linear regression plot showing the relationship between PC2 (exploration quality) and wayfinding success in the young group. Higher PC2 scores were associated with higher wayfinding success.
